# Two refined creep calculation methods of two-way prestressed concrete

**DOI:** 10.1371/journal.pone.0330075

**Published:** 2025-08-11

**Authors:** Pengfei Wu, Xinyu Xu, Zhichun Fang

**Affiliations:** 1 State Key Laboratory of Coastal and Offshore Engineering, Dalian University of Technology, Dalian, China; 2 Institute of Civil and Architectural Engineering, Tongling University, Tongling, China; Mirpur University of Science and Technology, PAKISTAN

## Abstract

With the advancement of engineering technology, prestressed concrete has been increasingly applied in various structures. To accurately and efficiently evaluate the long-term performance of prestressed concrete members, this paper proposes trapezoidal and difference methods for long-term deformation calculation based on the principle of creep superposition. Compared with existing creep refinement approaches and experimental data, the methods presented in this study demonstrate higher accuracy. Moreover, they significantly reduce computational complexity, offering a practical theoretical foundation for creep analysis in large-scale structures. These methods are further extended to two-way prestressed concrete members, addressing the engineering need for accurate long-term performance evaluation in such systems. The findings indicate that the creep development in two-way prestressed members is slower than that in one-way members.

## 1. Introduction

Prestressed concrete is widely used in bridges, ports, nuclear power plants, and other engineering structures. The effective prestress plays a crucial role in determining the structural performance [1–4]. However, due to shrinkage, creep, and prestress relaxation, the prestress gradually decreases over time [5–8]. This loss of prestress not only alters the internal stress distribution of the concrete but also affects the progression of creep. Therefore, in prestressed concrete structures, shrinkage, creep, and prestress relaxation are interdependent and interact with one another [9,10]. Accurate long-term deformation analysis must account for the coupling effects among these factors, rather than treating their contributions as separate and directly additive.

At present, most design codes for prestressed concrete in various countries adopt simplified calculation formulas [11–13]. For instance, the Code for Design of Highway Reinforced Concrete and Prestressed Concrete Bridges and Culverts (JTG 3362−2018) [14] considers the stress in reinforcement caused by concrete shrinkage as the total prestress loss due to both shrinkage and creep when calculating axial force and eccentricity. However, the code acknowledges that this approach introduces errors when the centroids of the prestressed and non-prestressed reinforcement do not coincide. Creep is the primary contributor to the long-term deformation of prestressed concrete. Despite this, the code does not employ a creep-based theoretical formulation for long-term deformation. Instead, it applies an empirical coefficient to the short-term deformation result, which limits the accuracy and calls for further improvement.

Current research on prestressed concrete primarily focuses on one-way prestressed members. However, with the advancement of engineering practices, the application of two-way prestressing has become increasingly widespread. For example, structures such as prestressed concrete box girders in long-span bridges and containment vessels in nuclear power plants often require two-way prestressed reinforcement [15]. In recent years, some scholars have explored two-way prestressed concrete [16,17], but these studies have mainly concentrated on its short-term performance. For long-term analysis, the calculation methods still rely on those developed for one-way prestressed members [18]. Existing research indicates that prestresses in different directions can influence each other. Therefore, to accurately evaluate the long-term behavior of two-way prestressed concrete members, it is essential to account for the interaction between prestresses in both directions.

To address these shortcomings, this chapter proposes a refined calculation method for long-term deformation that systematically considers the coupled effects of concrete shrinkage, creep, and prestress relaxation, significantly improving computational accuracy and theoretical consistency. For the coupling terms in the integral form of the equation, this chapter innovatively introduces two solution strategies: the trapezoidal method and the difference method, which not only enhance numerical stability but also expand the applicability of conventional methods. On this basis, the proposed method is further extended to the analysis of two-way prestressed concrete structures, and a long-term performance calculation method that accurately accounts for the interaction between bidirectional prestressing is developed, effectively overcoming the limitations of existing methods in complex stress systems.

### 2. Calculation method for long-term deformation of prestressed concrete beam

Shrinkage is an inherent property of concrete. Even in the absence of external loads, shrinkage can induce internal stresses within a structure [19–21]. When the cross-section is relatively small, concrete shrinkage can generally be considered uniform [22]. However, if the reinforcement is asymmetrically distributed, resulting in different constraints on the upper and lower parts of the section, uneven shrinkage may occur. Additionally, the restraining effect of the reinforcement introduces internal stresses in both the reinforcement and the surrounding concrete, which in turn triggers creep in the concrete. As creep develops, it gradually relieves the stresses caused by shrinkage. Therefore, even without external loading, shrinkage-induced deformation is inherently coupled with the creep process.

Creep calculation is a key method for predicting the additional deformation of concrete over time under long-term loading. Due to the viscoelastic nature of concrete, it undergoes time-dependent creep deformation when subjected to sustained stress. The calculation is typically based on the creep compliance function and the principle of superposition. The creep function represents the strain produced by a unit stress over time. Using the superposition principle, the incremental creep strains caused by loads applied at different times are accumulated to form an integral expression for the total strain or displacement. Since this integral is often complex, numerical methods such as the trapezoidal method and the difference method are commonly employed to solve it efficiently. Based on this theoretical framework, this chapter proposes improved versions of the trapezoidal and difference methods, enabling refined and efficient calculation of long-term creep deformation in prestressed concrete.

In this paper, the following assumptions are made: (1) The deformation meets the plane section assumption; (2) The concrete shrinkage shall be uniform; (3) The reinforcement and concrete are completely bonded, meeting the deformation coordination conditions; (4) The creep is linear, meeting Boltzman superposition principle; (5) Concrete is considered as homogeneous and isotropic material; (6) The reinforcement is always elastic; (7) The concrete is not cracked. Based on the static equilibrium, deformation compatibility and constitutive equation of static analysis, the expression of creep refinement method is derived in this paper.

### 2.1. Basic equation for long-term deformation calculation of prestressed concrete beam

According to the static equilibrium conditions, at any moment, the resultant force and moment of the concrete and reinforcement of the member section remain balanced:


$$\int_{0}^{h}\sigma_{c}\left(t,t_{s},y\right)b(y)dy+\sum_{k=1}^{n}\sigma_{s}\left(t,t_{s},y_{sk}\right)A_{sk}=0$$ (1)

$$\int_{0}^{h}\sigma_{c}\left(t,t_{s},y\right)b(y)ydy+\sum_{k=1}^{n}\sigma_{s}\left(t,t_{s},y_{sk}\right)A_{sk}y_{sk}=0$$ (2)

Where, $t_{s}$ is the time when the shrinkage starts. $\sigma_{c}\left(t,t_{s},y\right)$ is the stress of concrete at y from the upper edge at time t. $\sigma_{s}\left(t,t_{s},y_{sk}\right)$ is the stress of the reinforcement in row k of the section at time t. Reinforcement includes ordinary reinforcement and prestressed reinforcement. When k∈[1,m], it is ordinary reinforcement. When k∈(m+1,n], it is prestressed reinforcement. $y_{sk}$ is the distance from the gravity center of the reinforcement in row k to the upper edge. $A_{sk}$ is the area of reinforcement in row k.

**Fig 1:**
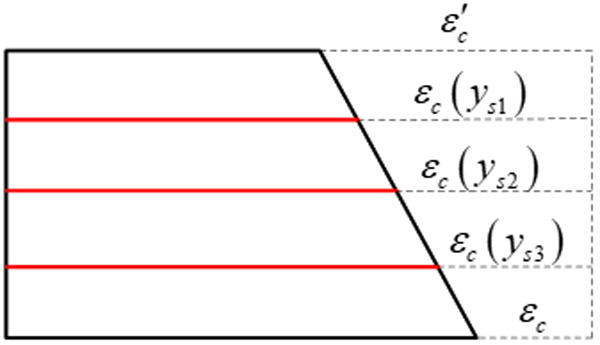
Section strain.

Since the member conforms to the plane section assumption, the concrete strain is linearly distributed along the height of the section, as shown in Fig 1.

From time $t_{s}$ when the concrete starts to shrink to time t, the strain at y from the upper edge of the section is:


$$\varepsilon_{c}\left(t,t_{s},y\right)=\varepsilon_{c}^{\prime }(t,t_{s})+\frac{y}{h}\left[\varepsilon_{c}(t,t_{s})-{\varepsilon^{\prime }}_{c}(t,t_{s})\right]$$ (3)

Where, $\varepsilon^{\prime }(t,t_{s})$ and $\varepsilon_{c}(t,t_{s})$ are the strains at the upper and lower edges of the section at time t. C is the section height. From this, it can be concluded that:


$$\varepsilon_{c}\left(t,t_{s},y_{sk}\right)=\varepsilon_{c}^{\prime }(t,t_{s})+\frac{y_{sk}}{h}\left[\varepsilon_{c}(t,t_{s})-{\varepsilon^{\prime }}_{c}(t,t_{s})\right]$$ (4)

Where, $\varepsilon_{c}\left(t,t_{s},y_{sk}\right)$ is the section strain at row k reinforcement.

As the stress-strain relationship of concrete conforms to the principle of creep superposition, the initial stress of concrete at $t_{s}$ is zero. Therefore, from $t_{s}$ to t, the strain at y from the upper edge is:


$$\varepsilon_{c}\left(t,t_{s},y\right)=\int_{t_{s}}^{t}J\left(t,\tau \right)d\sigma_{c}\left(\tau ,y\right)+\varepsilon_{sh}\left(t,t_{s}\right)$$ (5)

Where, $\sigma_{c}\left(\tau ,y\right)$ is the concrete stress at y from the upper edge. J(t,τ) is the creep compliance of concrete, $\varepsilon_{sh}\left(t,t_{s}\right)$ is the shrinkage strain of concrete, which can be calculated by methods in different codes. In this paper, the creep compliance and shrinkage strain of concrete are calculated by the method in fib MC 2020.

Since the reinforcement is always in elastic state, there are:


$$\sigma_{s}\left(t,t_{s},y_{sk}\right)=\varepsilon_{c}\left(t,t_{s},y_{sk}\right)E_{s}$$ (6)

Where, $\sigma_{s}\left(t,t_{s},y_{sk}\right)$ is the stress of reinforcement in row k. $\varepsilon_{c}\left(t,t_{s},y_{sk}\right)$ is the strain of reinforcement in row k. $E_{s}$ is the elastic modulus of reinforcement.

For prestressed reinforcement, there are:


$$\sigma_{p}\left(t,t_{s},y_{sk}\right)=\varepsilon_{c}\left(t,t_{s},y_{sk}\right)E_{s}+\chi \sigma_{pr}\left(t,t_{0}\right)$$ (7)

Where, $\sigma_{p}\left(t,t_{s},y_{sk}\right)$ is the stress of the k row prestressed reinforcement. χ is the relaxation reduction coefficient of prestressed reinforcement, which is taken as 0.8 in this paper. $\sigma_{pr}\left(t,t_{0}\right)$ is the relaxation stress of prestressed reinforcement. This paper adopts the trapezoidal method and the difference method for further calculation [23].

### 2.2. Trapezoid method for creep calculation

According to Eq (1), there are:


$$\int_{0}^{h}\int_{t_{s}}^{t}J\left(t,\tau \right)d\sigma_{c}\left(\tau ,t_{s},y\right)b(y)dy+\sum_{l=1}^{k}\int_{t_{s}}^{t}J\left(t,\tau \right)d\sigma_{s}\left(\tau ,t_{s},y_{sl}\right)A_{sl}=0$$ (8)

$$\int_{0}^{h}\left\{{\varepsilon^{\prime }}_{c}(t,t_{s})+\frac{y}{h}\left[\varepsilon_{c}(t,t_{s})-{\varepsilon^{\prime }}_{c}(t,t_{s})\right]\right\}b(y)dy+\sum_{l=1}^{k}\left[\varepsilon_{c}(t,t_{s},y_{sl})A_{sl}\right]=\int_{0}^{h}\varepsilon_{sh}\left(t,t_{s}\right)b(y)dy$$ (9)

After sorting out:


$$\begin{array}{c} \int_{0}^{h}\left(1-\frac{y}{h}\right)b\left(y\right)dy{\varepsilon^{\prime }}_{c}(t,t_{s})+\int_{0}^{h}\frac{y}{h}b\left(y\right)dy\varepsilon_{c}(t,t_{s})+\sum_{l=1}^{k}\left[\varepsilon_{c}(t,t_{s},y_{sl})A_{sl}\right]\\ =\int_{0}^{h}\varepsilon_{sh}\left(t,t_{s}\right)b(y)dy \end{array}$$ (10)

According to Eq (2), there are:


$$\int_{0}^{h}\int_{t_{s}}^{t}J\left(t,\tau \right)d\sigma_{c}\left(\tau ,t_{s},y\right)b(y)ydy+\sum_{l=1}^{k}\int_{t_{s}}^{t}J\left(t,\tau \right)d\sigma_{s}\left(\tau ,t_{s},y_{sl}\right)A_{sl}y_{sl}=0$$ (11)

$$\begin{array}{c} \int_{0}^{h}\left\{{\varepsilon^{\prime }}_{c}(t,t_{s})+\frac{y}{h}\left[\varepsilon_{c}(t,t_{s})-{\varepsilon^{\prime }}_{c}(t,t_{s})\right]\right\}b(y)ydy+\sum_{l=1}^{k}\left[\varepsilon_{c}(t,t_{s},y_{sl})A_{sl}y_{sl}\right]\\ =\int_{0}^{h}\varepsilon_{sh}\left(t,t_{s}\right)b(y)ydy \end{array}$$ (12)

After sorting out:


$$\begin{array}{c} \int_{0}^{h}\left(1-\frac{y}{h}\right)b\left(y\right)ydy{\varepsilon^{\prime }}_{c}(t,t_{s})+\int_{0}^{h}\frac{y}{h}b\left(y\right)ydy\varepsilon_{c}(t,t_{s})+\sum_{l=1}^{k}\left[\varepsilon_{c}(t,t_{s},y_{sl})A_{sl}y_{sl}\right]\\ =\int_{0}^{h}\varepsilon_{sh}\left(t,t_{s}\right)b(y)ydy \end{array}$$ (13)

According to Eq (4), there are:


$$\left(1-\frac{y_{sk}}{h}\right)\varepsilon_{c}^{\prime }(t_{n},t_{s})+\frac{y_{sk}}{h}\varepsilon_{c}(t_{n},t_{s})-\varepsilon_{c}\left(t_{n},t_{s},y_{sk}\right)=0$$ (14)

According to Eq (6), there are:


$$\sigma_{s}\left(t,t_{s},y_{sk}\right)-\varepsilon_{s}\left(t,t_{s},y_{sk}\right)E_{s}=0$$ (15)

$$\sigma_{p}\left(t,t_{s},y_{sk}\right)-\varepsilon_{c}\left(t,t_{s},y_{sk}\right)E_{s}=\chi \sigma_{pr}\left(t,t_{0}\right)$$ (16)

The integral term $\int_{t_{s}}^{t}J\left(t,\tau \right)d\sigma_{c}\left(\tau ,y\right)$ exists in Eq (5). As the stress of concrete changes with time during creep, Eq (5) cannot be directly integrated. In order to solve the equations, the trapezoid method and difference method are proposed in this paper. Order:


$$f\left(\tau \right)=J\left(t,\tau \right)d\sigma_{c}\left(\tau ,y\right)$$ (17)

Then:


$$\int_{t_{s}}^{t}J\left(\tau \right)d\sigma_{c}\left(\tau ,y\right)=\int_{t_{s}}^{t}f\left(\tau \right)d\tau$$ (18)

At this time, the integral term of Eq (18) can be expressed as:


$$\int_{t_{s}}^{t_{i}}f\left(\tau \right)d\tau =\sum_{j=1}^{i}\left\{\frac{1}{2}\Delta t_{j}\left[f\left(t_{j}\right)+f\left(t_{j-1}\right)\right]\right\}$$ (19)

Eq (5) can be discretized as:


$$\varepsilon_{c}\left(t_{i},t_{s},y\right)=\sum_{j=1}^{i}\left\{\frac{1}{2}\left[\sigma_{c}\left(\tau_{j},y\right)-\sigma_{c}\left(\tau_{j-1},y\right)\mathrm{\backslash rightleft}[J\left(t_{i},t_{j}\right)+J\left(t_{i},t_{j-1}\right)\right]\right\}+\varepsilon_{sh}\left(t_{i},t_{s}\right)$$ (20)

After sorting out:


$$\begin{array}{c} \varepsilon_{c}\left(t_{i},t_{s},y_{sk}\right)-\frac{1}{2}\sigma_{c}\left(t_{i},y_{sk}\right)[J\left(t_{i},t_{i-1}\right)+J\left(t_{i},t_{i}\right)\backslash =-\frac{1}{2}\sigma_{c}\left(t_{i-1},y_{sk}\right)\left[J\left(t_{i},t_{i-1}\right)+J\left(t_{i},t_{i}\right)\right]\\ +\sum_{j=1}^{i-1}\left\{\frac{1}{2}\left[\sigma_{c}\left(t_{i},y_{sk}\right)-\sigma_{c}\left(t_{i-1},y_{sk}\right)\right]\left[J\left(t_{i},t_{j-1}\right)+J\left(t_{i},t_{j}\right)\right]\right\}+\varepsilon_{sh}\left(t_{i},t_{s}\right) \end{array}$$ (21)

The greater the number of discrete points, the higher the calculation accuracy, but the computational efficiency will decrease accordingly. If the entire creep process is discretized uniformly over all time intervals, the resulting computational load would be excessive. Considering that creep develops rapidly in the early stage and gradually stabilizes in the later stage, this paper adopts a non-uniform discretization strategy: denser discrete points are used in the early stage of creep, while sparser points are applied in the later stage. This approach ensures sufficient accuracy in the early stage while significantly reducing the overall computational resource consumption.The discrete method of time period is:


$$t_{j}=\frac{1}{2^{i-j}}\left(t_{i}-t_{s}\right)+t_{s},j=1,2\cdots i$$ (22)

Order:


$$\begin{array}{c} A_{1}=\left(\begin{array}{cccc} {}*20ca_{11} & a_{12} & \cdots & a_{1k}\\ a_{21} & a_{22} & \cdots & a_{2k} \end{array}\right),A_{2}=\left(\begin{array}{cc} {}*20cc_{11} & c_{12}\\ c_{21} & c_{22} \end{array}\right)\\ A_{3}=\left(\begin{array}{cc} {}*20c\frac{y_{s1}}{h} & 1-\frac{y_{s1}}{h}\\ \frac{y_{s2}}{h} & 1-\frac{y_{s2}}{h}\\ \cdots & \cdots \\ \frac{y_{sk}}{h} & 1-\frac{y_{sk}}{h} \end{array}\right),A_{4}={\left(\begin{array}{cccc} {}*20c-E_{s} & 0 & \cdots & 0\\ 0 & -E_{s} & \cdots & 0\\ \cdots & \cdots & \cdots & \cdots \\ 0 & 0 & \cdots & -E_{s} \end{array}\right)}_{k\times k}\\ A_{5}={\left(\begin{array}{cccc} {}*20cd & 0 & \cdots & 0\\ 0 & d & \cdots & 0\\ \cdots & \cdots & \cdots & \cdots \\ 0 & 0 & \cdots & d \end{array}\right)}_{k\times k},A_{6}=E_{k\times k} \end{array}$$ (23)

$$\begin{array}{c} x_{1}={\left(\begin{array}{c} {}*20c\sigma_{s}\left(t_{i},t_{s},y_{s1}\right)\\ \sigma_{s}\left(t_{i},t_{s},y_{s2}\right)\\ \cdots \\ \sigma_{s}\left(t_{i},t_{s},y_{sk}\right) \end{array}\right)}_{k\times 1},x_{2}={\left(\begin{array}{c} {}*20c\sigma_{c}\left(t_{i},t_{s},y_{s1}\right)\\ \sigma_{c}\left(t_{i},t_{s},y_{s2}\right)\\ \cdots \\ \sigma_{c}\left(t_{i},t_{s},y_{sk}\right) \end{array}\right)}_{k\times 1}\\ x_{3}={\left(\begin{array}{c} {}*20c\varepsilon_{c}\left(t_{i},t_{s},y_{s1}\right)\\ \varepsilon_{c}\left(t_{i},t_{s},y_{s2}\right)\\ \cdots \\ \varepsilon_{c}\left(t_{i},t_{s},y_{sk}\right) \end{array}\right)}_{k\times 1},x_{4}=\left(\begin{array}{c} {}*20c\varepsilon_{c}\left(t_{i},t_{s}\right)\\ {\varepsilon^{\prime }}_{c}\left(t_{i},t_{s}\right) \end{array}\right) \end{array}$$ (24)

$$\begin{array}{c} b_{1}=\left(\begin{array}{c} {}*20cl_{1}\\ l_{2} \end{array}\right)\\ b_{2}=\left(\begin{array}{c} {}*20c0_{m\times 1}\\ \chi \sigma_{pr}\left(t,t_{0},y_{s\left(m+1\right)}\right)\\ \chi \sigma_{pr}\left(t,t_{0},y_{s\left(m+2\right)}\right)\\ \cdots \\ \chi \sigma_{pr}\left(t,t_{0},y_{sk}\right) \end{array}\right)\\ b_{3}=\left(\begin{array}{c} {}*20cm_{1}\\ m_{2}\\ \cdots \\ m_{k} \end{array}\right) \end{array}$$ (25)

Where,


$$\begin{array}{c} a_{1l}=A_{sl}\\ a_{2l}=A_{sl}y_{sl},l=1,2\cdots k \end{array}$$ (26)

$$\begin{array}{c} c_{11}=\int_{0}^{h}\frac{y}{h}b\left(y\right)dy\\ c_{12}=\int_{0}^{h}\left(1-\frac{y}{h}\right)b\left(y\right)dy\\ c_{21}=\int_{0}^{h}\frac{y}{h}b\left(y\right)ydy\\ c_{22}=\int_{0}^{h}\left(1-\frac{y}{h}\right)b\left(y\right)ydy \end{array}$$ (27)

$$d=-\frac{1}{2}\left[J\left(t_{i},t_{i}\right)+J\left(t_{i},t_{i-1}\right)\right]$$ (28)

$$\begin{array}{c} l_{1}=\int_{0}^{h}\varepsilon_{sh}\left(t,t_{s}\right)b(y)dy\\ l_{2}=\int_{0}^{h}\varepsilon_{sh}\left(t,t_{s}\right)b(y)ydy \end{array}$$ (29)

$$\begin{array}{c} m_{l}=-\frac{1}{2}\sigma_{c}\left(t_{n-1},y_{sl}\right)[J\left(t_{i},t_{i-1}\right)+J\left(t_{i},t_{i}\right)\backslash +\sum_{j=1}^{i-1}\left\{\frac{1}{2}\left[\sigma_{c}\left(t_{j},y_{sl}\right)-\sigma_{c}\left(t_{j-1},y_{sl}\right)\right]\left[J\left(t_{i},t_{j-1}\right)+J\left(t_{i},t_{j}\right)\right]\right\}+\varepsilon_{sh}\left(t_{i},t_{s}\right),l=1,2\cdots k \end{array}$$ (30)

Then the creep expression of trapezoidal method can be expressed as:


Ax=b (31)

Where,


$$A=\left(\begin{array}{cccc} {}*20c0 & 0 & A_{1} & A_{2}\\ 0 & 0 & -E_{k\times k} & A_{3}\\ E_{k\times k} & 0 & A_{4} & 0\\ 0 & A_{5} & A_{6} & 0 \end{array}\right)$$ (32)

$$x=\left(\begin{array}{c} {}*20cx_{1}\\ x_{2}\\ x_{3}\\ x_{4} \end{array}\right)$$ (33)

$$b=\left(\begin{array}{c} {}*20cb_{1}\\ 0\\ b_{2}\\ b_{3} \end{array}\right)$$ (34)

The solution of the equation is:


$$x=A^{-1}b$$ (35)

In the matrix of the equations, $A_{1},A_{2}$ represents the stress state of the structure. $b_{1}$ represents the stress boundary condition of the structure. $A_{3}$ represents the composition of reinforcement. $A_{4},A_{5}$ represents the constitutive condition of the reinforcement. $A_{6},A_{7}$ represents the calculation method of creep. $b_{2}$ is the boundary condition of creep calculation method. When the calculation conditions change, it is only necessary to adjust the corresponding sub matrix without re deriving the entire equation, which provides convenience for engineering calculation under different conditions.

### 2.3. Difference method for creep calculation

The trapezoidal method needs to store the information of the intermediate calculation process of each time step, which has certain disadvantages in the creep analysis of large and complex structures. Therefore, some scholars have studied the creep calculation method based on the viscoelastic model. When using the viscoelastic model calculation method, there is no need to store the intermediate calculation information of each time step, which saves a certain amount of calculation [24]. However, the parameters of viscoelastic model can not be determined according to the composition and mechanical properties of materials at present. Generally, the parameters in the model are fitted according to the known creep equation. The fitted model parameters often have some limitations in application. If the initial loading time is short, the creep calculation results based on viscoelastic model are not ideal [24,25]. Therefore, the difference method for creep calculation is proposed in this paper. The partial derivative of Eq (5) gives:


$$\frac{\partial \varepsilon_{c}\left(t,t_{s},y\right)}{\partial \sigma_{c}\left(\tau ,y\right)}=J\left(t,\tau \right)$$ (36)

The boundary conditions of the differential equation is:


$$\varepsilon_{c}\left(t_{j},t_{s},y\right)=\varepsilon_{c}\left(t_{j-1},t_{s},y\right)+\varepsilon_{sh}\left(t_{j},t_{s}\right)-\varepsilon_{sh}\left(t_{j-1},t_{s}\right)$$ (37)

The Euler method [26] is used for the difference of Eq (36) to obtain:


$$\varepsilon_{c}\left(t_{j},t_{s},y\right)=\varepsilon_{c}\left(t_{j-1},t_{s},y\right)+\left[\sigma_{c}\left(t_{j},t_{s},y\right)-\sigma_{c}\left(t_{j-1},t_{s},y\right)\right]J\left(t_{i},t_{i}\right)$$ (38)

After sorting out:


$$\varepsilon_{c}\left(t_{i},t_{s},y\right)-J\left(t_{i},t_{i}\right)\sigma_{c}\left(t_{i},y\right)=\varepsilon_{c}\left(t_{i-1},t_{s},y\right)-J\left(t_{i},t_{i}\right)\sigma_{c}\left(t_{i-1},y\right)+\varepsilon_{sh}\left(t_{i},t_{s}\right)$$ (39)

As the stress conditions of the structure and the composition of the reinforcement remain unchanged, only the calculation method has changed, so only $A_{5},A_{6},b_{3}$ needs to be adjusted, and the expression is:


$$A_{5}={\left(\begin{array}{cccc} {}*20c-J\left(t_{i},t_{i}\right) & 0 & \cdots & 0\\ 0 & -J\left(t_{i},t_{i}\right) & \cdots & 0\\ \cdots & \cdots & \cdots & \cdots \\ 0 & 0 & \cdots & -J\left(t_{i},t_{i}\right) \end{array}\right)}_{k\times k},A_{6}=E_{k\times k}$$ (40)

$$m_{l}=\varepsilon_{c}\left(t_{i-1},t_{s},y_{sl}\right)-J\left(t_{i},t_{i}\right)\sigma_{c}\left(t_{i-1},y_{sl}\right)+\varepsilon_{sh}\left(t_{i},t_{s}\right),l=1,2\cdots k$$ (41)

The other forms remain unchanged and can still be solved by Eq (35).

## 3. Example analysis

**Fig 2:**
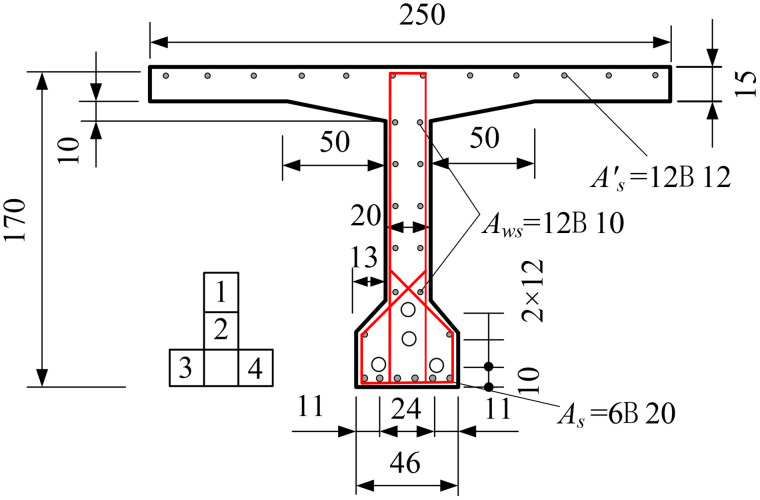
Beam size and reinforcement (Unit: cm).

In order to verify the accuracy of the method in this paper, a T-beam is taken as an example. The beam is designed according to the section in reference [27]. The beam is made of C40 concrete. The prestressed steel strand is made of 4 strands of high-strength low relaxation 7-wire twisted prestressed steel strand, with a diameter of 15.20 mm, a area of 140mm^2^, a standard strength of $f_{pk}=1860MPa$, and an elastic modulus of $E_{p}=1.95\times 10^{5}MPa$. The gravity center of the prestressed steel strand is 190 mm from the lower edge of the section. HRB335 is adopted for ordinary reinforcement, the standard value of tensile strength is $f_{sk}=335MPa$, and the elastic modulus is $E_{s}=2.0\times 10^{5}MPa$. The distance from the gravity center of upper, lower and web reinforcement to the lower edge is 1660 mm, 900 mm and 40 mm respectively. The specific section is shown in Fig 2.

**Fig 3:**
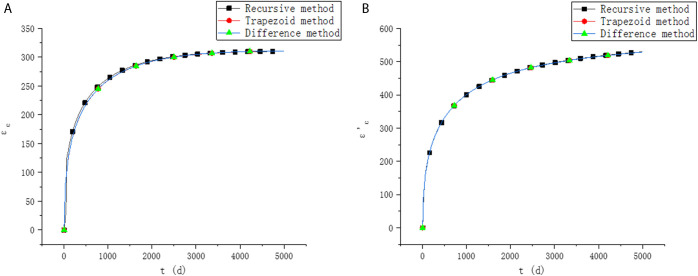
Comparison of strain results. (a) Lower edge strain. (b) Upper edge strain.

**Fig 4:**
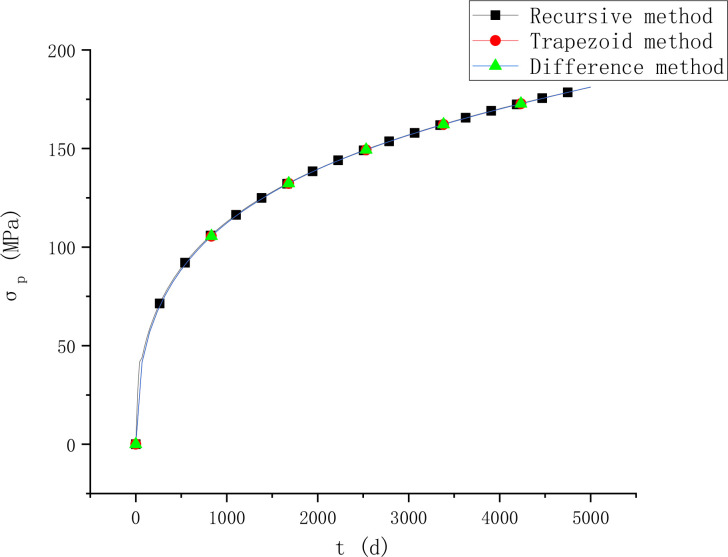
Comparison of prestress loss results.

It is assumed that the relative humidity of the environment is 60%. The shrinkage and creep model is adopted as methods in fib MC 2010. For the calculation of creep, reference [28] proposed a recursive method, but the recursive method has a large calculation amount and is more used for the verification of creep method. In order to verify the accuracy of the method in this paper, the results in this paper are compared with that by the recursive method. The upper edge strain $\varepsilon_{c}^{\prime }$, lower edge strain $\varepsilon_{c}$ and prestress loss $\sigma_{p}$ under different methods are shown in Figs 3 and 4.

**Fig 5:**
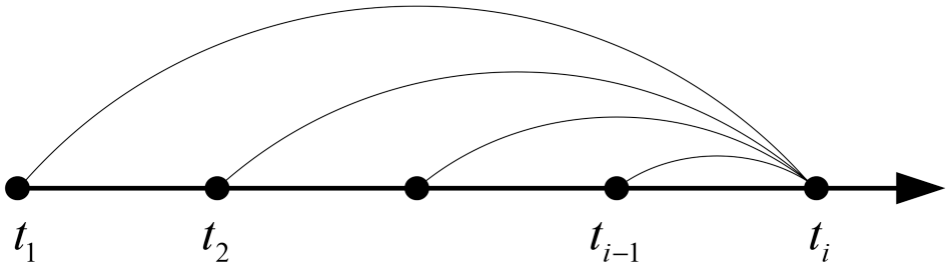
Calculation steps of recursive method and trapezoid method.

It can be seen from Figs 3 and 4 that the results of the two methods proposed in this paper are similar, and both are in good agreement with the results of existing methods, which verifies the accuracy of the methods in this paper. When the recursive method and trapezoidal method are used to calculate the stress and strain at time $t_{i}$, the stress and strain at $t_{1},t_{2},\cdots t_{i-1}$ need to be considered, as shown in Fig 5.

For the recursive method, the calculation steps required to calculate to time $t_{i}$ are:


$$n=1+2+\cdots +i=\frac{i\left(i+1\right)}{2}$$ (42)

For the trapezoidal method, although the calculation steps are similar to those of the recursive method, the method in this paper only needs to disperse the time into a few periods to obtain higher accuracy. The trapezoidal method discretizes the time into 8 periods in Figs 3 and 4. The calculation steps required by the trapezoidal method are:


n=8i (43)

**Fig 6:**
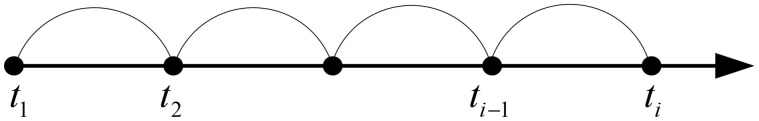
Calculation steps of difference method.

When using the difference method to calculate the stress and strain at time $t_{i}$, it is unnecessary to consider the stress and strain at time $t_{1},t_{2},\cdots t_{i-2}$. The calculation result at time $t_{i}$ can be obtained from time $t_{i-1}$, as shown in Fig 6.

Therefore, the calculation steps required for the difference method to calculate to time $t_{i}$ are:


n=i (44)

**Fig 7:**
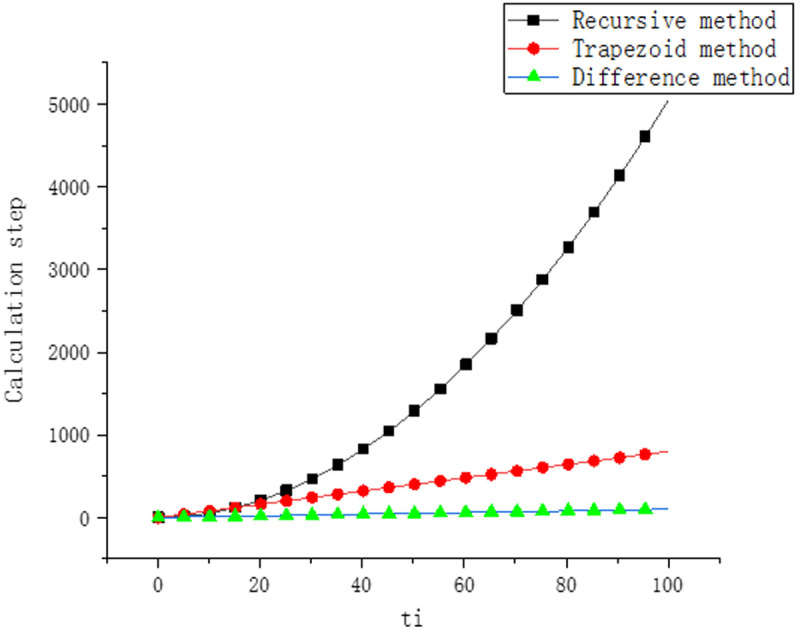
Calculation steps of different method.

It can be seen from Eq (42)–(44) that the calculation steps required by the recursive method show a quadratic increasing law with the increase of time. The calculation steps required by trapezoidal method and difference method increase linearly with time, as shown in Fig 7.

Therefore, with the increase of calculation time A, the gap of calculation efficiency between the methods in this paper and that of the recursive method will become larger and larger. The methods in this paper can not only ensure the calculation accuracy, but also greatly improve the calculation efficiency.

In order to further verify the accuracy of the methods in this paper, experimental data in reference [29] are collected. The experimental results are compared with the calculated results in this paper. Reference [25] has conducted an experimental study on 8 prestressed concrete beams (PC1 ~ PC8). The concrete strength grade is C55. The section size is 200 × 300 mm. The calculated span is 4200 mm. The reinforcement information is shown in Table 1. During the experiment, the temperature varies from 4°C to 35°C, and the average temperature is 18°C. The variation range of humidity is 43% ~ 98%, and the average humidity is 73%.

**Table 1:** Beam reinforcement information.

Beam	Prestressed reinforcement	Distance from prestressed reinforcement to upper edge(mm)	Initial tension of prestressed reinforcement(kN)	Area of upper reinforcement(mm^2^)	Distance from upper reinforcement to upper edge(mm)	Area of lower reinforcement(mm^2^)	Distance from lower reinforcement to upper edge(mm)
PC1	1Φ15.2	240	100.1	226	40	339	260
PC2	1Φ15.2	240	103.9	226	40	339	260
PC3	1Φ15.2	240	102.3	226	40	339	260
PC4	1Φ15.2	240	135.9	226	40	339	260
PC5	1Φ15.2	240	108.7	226	40	339	260
PC6	2Φ15.2	60/240	101.4	226	40	339	260
PC7	1Φ15.2	240	102.6	402	40	603	260
PC8	1Φ15.2	240	106.0	226	40	339	260

**Fig 8:**
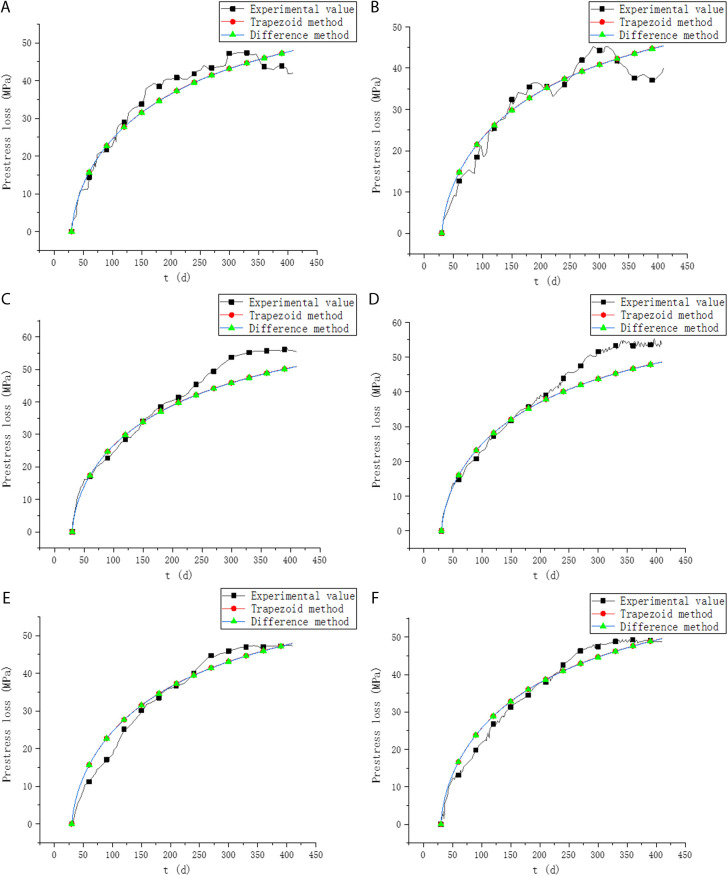
Comparison of prestress loss. (a) PC1. (b) PC3. (c) PC5. (d) PC6. (e) PC7. (f) PC8.

Since PC2 is unbonded prestress, PC4 does not give prestress loss results in the original paper, this paper gives a comparison between the experimental and theoretical values of prestress loss of the remaining six beams, as shown in Fig 8.

It can be seen from Fig 8 and Table 2 that the two methods in this paper are in good agreement with the experimental results. By comparing the results of this paper with the experimental results, it is shown that the methods in this paper can ensure the accuracy, which can be used as one of the effective analysis methods for the long-term performance of prestressed concrete. However, due to the presence of numerous complex physicochemical reactions during the creep process and the continuous variation of daily environmental factors, certain discrepancies in the calculation results may occur.

**Table 2:** RMSE.

	Trapezoid method	Difference method
PC1	2.941	2.967
PC3	3.500	3.491
PC5	4.473	4.518
PC6	4.578	4.616
PC7	2.746	2.741
PC8	2.433	2.436

Its Root Mean Squared Error (RMSE) is shown in Table 2.

## 4. Calculation method for long-term deformation of two-way prestressed beams

### 4.1. Basic equation for long-term deformation calculation of two-way prestressed beam

With the development of technology, two-way prestressed are more and more used in engineering. The two-way prestressed concrete is affected by the Poisson’s ratio, and the creep in both directions has a coupling effect. Some studies show that [30], when the concrete is loaded in two directions for a long time, the Poisson’s ratio is not much different from that of one-way loading. In creep analysis, it can be considered that the Poisson’s ratio is not affected by creep.

For two-way prestressed concrete, due to the Poisson effect, the constitutive equation of concrete is:


$$\begin{array}{c} \varepsilon_{cx}\left(t,t_{s},y\right)=\int_{t_{s}}^{t}J\left(t,\tau \right)d\left[\sigma_{cx}\left(\tau ,y\right)-\mu \sigma_{cy}\left(\tau ,y\right)\right]+\varepsilon_{sh}\left(t,t_{s}\right)\\ \varepsilon_{cy}\left(t,t_{s},y\right)=\int_{t_{s}}^{t}J\left(t,\tau \right)d\left[\sigma_{cy}\left(\tau ,y\right)-\mu \sigma_{cx}\left(\tau ,y\right)\right]+\varepsilon_{sh}\left(t,t_{s}\right) \end{array}$$ (45)

Where, μ is the Poisson’s ratio.

Since there are prestressed reinforcement in two directions, it is necessary to expand the sub matrix into the form of two directions:


$$\begin{array}{cc} & {𝐀}_{1}=\left(\begin{array}{cccccccc} a_{11x} & a_{12x} & \cdots & a_{1kx} & 0 & 0 & \cdots & 0\\ a_{21x} & a_{22x} & \cdots & a_{2kx} & 0 & 0 & \cdots & 0\\ 0 & 0 & \cdots & 0 & a_{11y} & a_{12y} & \cdots & a_{1ky}\\ 0 & 0 & \cdots & 0 & a_{21y} & a_{22y} & \cdots & a_{2ky} \end{array}\right),{𝐀}_{2}=\left(\begin{array}{cccc} c_{11x} & c_{12x} & 0 & 0\\ c_{21x} & c_{22x} & 0 & 0\\ 0 & 0 & c_{11y} & c_{12y}\\ 0 & 0 & c_{21y} & c_{22y} \end{array}\right)\\ & {𝐀}_{3}=\left(\begin{array}{cccc} \frac{y_{s1x}}{h} & 1-\frac{y_{s1x}}{h} & 0 & 0\\ \frac{y_{s2x}}{h} & 1-\frac{y_{s2x}}{h} & 0 & 0\\ \cdots & \cdots & \cdots & \cdots \\ \frac{y_{skx}}{h} & 1-\frac{y_{skx}}{h} & 0 & 0\\ 0 & 0 & \frac{y_{ys1y}}{h} & 1-\frac{y_{ys1y}}{h}\\ 0 & 0 & \frac{y_{ys2y}}{h} & 1-\frac{y_{ys2y}}{h}\\ \cdots & \cdots & \cdots & \cdots \\ 0 & 0 & \frac{y_{sky}}{h} & 1-\frac{y_{sky}}{h} \end{array}\right),{𝐀}_{4}={\left(\begin{array}{cccc} -E_{s} & 0 & \cdots & 0\\ 0 & -E_{s} & \cdots & 0\\ \cdots & \cdots & \cdots & \cdots \\ 0 & 0 & \cdots & -E_{s} \end{array}\right)}_{\left(kx+ky\right)\times \left(kx+ky\right)}\\ & {𝐀}_{5}={\left(\begin{array}{cccccccc} d_{1} & 0 & \cdots & 0 & d_{2} & d_{2} & \cdots & d_{2}\\ 0 & d_{1} & \cdots & 0 & d_{2} & d_{2} & \cdots & d_{2}\\ \cdots & \cdots & \cdots & \cdots & \cdots & \cdots & \cdots & \cdots \\ 0 & 0 & \cdots & d_{1} & d_{2} & d_{2} & \cdots & d_{2}\\ d_{2} & d_{2} & \cdots & d_{2} & d_{1} & 0 & \cdots & 0\\ d_{2} & d_{2} & \cdots & d_{2} & 0 & d_{1} & \cdots & 0\\ \cdots & \cdots & \cdots & \cdots & \cdots & \cdots & \cdots & \cdots \\ d_{2} & d_{2} & \cdots & d_{2} & 0 & 0 & \cdots & d_{1} \end{array}\right)}_{\left(kx+ky\right)\times \left(kx+ky\right)},{𝐀}_{6}={𝐄}_{\left(kx+ky\right)\times \left(kx+ky\right)} \end{array}$$ (46)

$$\begin{array}{cc} & {𝐱}_{1}=\left(\begin{array}{c} \sigma_{s}\left(t_{i},t_{\text{s}},y_{\text{s1x}}\right)\\ \sigma_{s}\left(t_{i},t_{\text{s}},y_{\text{s2x}}\right)\\ \cdots \\ \sigma_{s}\left(t_{i},t_{\text{s}},y_{\text{skx}}\right)\\ \sigma_{s}\left(t_{i},t_{\text{s}},y_{\text{s1y}}\right)\\ \sigma_{s}\left(t_{i},t_{\text{s}},y_{\text{s2y}}\right)\\ \cdots \\ \sigma_{s}\left(t_{i},t_{\text{s}},y_{\text{sky}}\right) \end{array}\right),{𝐱}_{2}=\left(\begin{array}{c} \sigma_{c}\left(t_{i},t_{\text{s}},y_{\text{s1x}}\right)\\ \sigma_{c}\left(t_{i},t_{\text{s}},y_{\text{s2x}}\right)\\ \cdots \\ \sigma_{c}\left(t_{i},t_{\text{s}},y_{\text{skx}}\right)\\ \sigma_{c}\left(t_{i},t_{\text{s}},y_{\text{s1y}}\right)\\ \sigma_{c}\left(t_{i},t_{\text{s}},y_{\text{s2y}}\right)\\ \cdots \\ \sigma_{c}\left(t_{i},t_{\text{s}},y_{\text{sky}}\right) \end{array}\right)\\ & {𝐱}_{3}=\left(\begin{array}{c} \varepsilon_{c}\left(t_{i},t_{\text{s}},y_{\text{s1x}}\right)\\ \varepsilon_{c}\left(t_{i},t_{\text{s}},y_{\text{s2x}}\right)\\ \cdots \\ \varepsilon_{c}\left(t_{i},t_{\text{s}},y_{\text{skx}}\right)\\ \varepsilon_{c}\left(t_{i},t_{\text{s}},y_{\text{s1y}}\right)\\ \varepsilon_{c}\left(t_{i},t_{\text{s}},y_{\text{s2y}}\right)\\ \cdots \\ \varepsilon_{c}\left(t_{i},t_{\text{s}},y_{\text{sky}}\right) \end{array}\right),{𝐱}_{4}=\left(\begin{array}{c} \varepsilon_{cx}\left(t_{i},t_{\text{s}}\right)\\ {\varepsilon^{\prime }}_{cx}\left(t_{i},t_{\text{s}}\right)\\ \varepsilon_{cy}\left(t_{i},t_{\text{s}}\right)\\ {\varepsilon^{\prime }}_{cy}\left(t_{i},t_{\text{s}}\right) \end{array}\right) \end{array}$$ (47)

$$\begin{array}{c} b_{1}=\left(\begin{array}{c} {}*20cl_{1x}\\ l_{2x}\\ l_{1y}\\ l_{2y} \end{array}\right)\\ b_{2}=\left(\begin{array}{c} {}*20c0_{mx\times 1}\\ \chi \sigma_{pr}\left(t,t_{0},y_{s\left(mx+1\right)}\right)\\ \chi \sigma_{pr}\left(t,t_{0},y_{s\left(mx+2\right)}\right)\\ \cdots \\ \chi \sigma_{pr}\left(t,t_{0},y_{skx}\right)\\ 0_{my\times 1}\\ \chi \sigma_{pr}\left(t,t_{0},y_{s\left(my+1\right)}\right)\\ \chi \sigma_{pr}\left(t,t_{0},y_{s\left(my+2\right)}\right)\\ \cdots \\ \chi \sigma_{pr}\left(t,t_{0},y_{sky}\right) \end{array}\right)\\ b_{3}=\left(\begin{array}{c} {}*20cm_{1x}\\ m_{2x}\\ \cdots \\ m_{kx}\\ m_{1y}\\ m_{2y}\\ \cdots \\ m_{ky} \end{array}\right) \end{array}$$ (48)

Where,


$$\begin{array}{c} a_{x1l}=A_{xsl}\\ a_{x2l}=A_{xsl}y_{xsl},l=1,2\cdots kx\\ a_{y1l}=A_{ysl}\\ a_{y2l}=A_{ysl}y_{ysl},l=1,2\cdots ky \end{array}$$ (49)

$$\begin{array}{cc} & c_{x11}=\int_{0}^{h}\frac{y}{h}b_{x}\left(y\right)\text{d}y+\mu \int_{0}^{h}\frac{y}{h}b_{y}\left(y\right)\text{d}y\\ & c_{x12}=\int_{0}^{h}\left(1-\frac{y}{h}\right)b_{x}\left(y\right)\text{d}y+\mu \int_{0}^{h}\left(1-\frac{y}{h}\right)b_{y}\left(y\right)\text{d}y\\ & c_{x21}=\int_{0}^{h}\frac{y}{h}b_{x}\left(y\right)y\text{d}y+\mu \int_{0}^{h}\frac{y}{h}b_{y}\left(y\right)y\text{d}y\\ & c_{x22}=\int_{0}^{h}\left(1-\frac{y}{h}\right)b_{x}\left(y\right)y\text{d}y+\mu \int_{0}^{h}\left(1-\frac{y}{h}\right)b_{y}\left(y\right)y\text{d}y\\ & c_{y11}=\int_{0}^{h}\frac{y}{h}b_{y}\left(y\right)\text{d}y+\mu \int_{0}^{h}\frac{y}{h}b_{x}\left(y\right)\text{d}y\\ & c_{y12}=\int_{0}^{h}\left(1-\frac{y}{h}\right)b_{y}\left(y\right)\text{d}y+\mu \int_{0}^{h}\left(1-\frac{y}{h}\right)b_{x}\left(y\right)\text{d}y\\ & c_{y21}=\int_{0}^{h}\frac{y}{h}b_{y}\left(y\right)y\text{d}y+\mu \int_{0}^{h}\frac{y}{h}b_{x}\left(y\right)y\text{d}y\\ & c_{y22}=\int_{0}^{h}\left(1-\frac{y}{h}\right)b_{y}\left(y\right)y\text{d}y+\mu \int_{0}^{h}\left(1-\frac{y}{h}\right)b_{x}\left(y\right)y\text{d}y \end{array}$$ (50)

$$\begin{array}{c} l_{1x}=\int_{0}^{h}\varepsilon_{sh}\left(t,t_{s}\right)b_{x}(y)dy\\ l_{2x}=\int_{0}^{h}\varepsilon_{sh}\left(t,t_{s}\right)b_{x}(y)ydy\\ l_{1y}=\int_{0}^{h}\varepsilon_{sh}\left(t,t_{s}\right)b_{y}(y)dy\\ l_{2y}=\int_{0}^{h}\varepsilon_{sh}\left(t,t_{s}\right)b_{y}(y)ydy \end{array}$$ (51)

### 4.2. Trapezoid method for creep calculation

Eq (45) is discretized as:


$$\begin{array}{c} \varepsilon_{cx}\left(t_{i},t_{s},y_{x}\right)=\sum_{j=1}^{i}\left\{\frac{1}{2}\left\{\left[\sigma_{c}\left(\tau_{j},y_{x}\right)-\mu \sigma_{c}\left(\tau_{j},y_{y}\right)\right]-\left[\sigma_{c}\left(\tau_{j-1},y_{x}\right)-\mu \sigma_{c}\left(\tau_{j-1},y_{y}\right)\right]\right\}\left[J\left(t_{i},t_{j}\right)+J\left(t_{i},t_{j-1}\right)\right]\right\}\\ \;\;\;\;\;\;\;\;\;\;\;\;\;\;\;\;\;\;\;\;\;\;\;\;\;\;\;\;+\varepsilon_{sh}\left(t_{i},t_{s}\right)\\ \varepsilon_{cy}\left(t_{i},t_{s},y_{y}\right)=\sum_{j=1}^{i}\left\{\frac{1}{2}\left\{\left[\sigma_{c}\left(\tau_{j},y_{y}\right)-\mu \sigma_{c}\left(\tau_{j},y_{x}\right)\right]-\left[\sigma_{cy}\left(\tau_{j-1},y_{y}\right)-\mu \sigma_{c}\left(\tau_{j-1},y_{x}\right)\right]\right\}\left[J\left(t_{i},t_{j}\right)+J\left(t_{i},t_{j-1}\right)\right]\right\}\\ \;\;\;\;\;\;\;\;\;\;\;\;\;\;\;\;\;\;\;\;\;\;\;\;\;\;\;\;\;+\varepsilon_{sh}\left(t_{i},t_{s}\right) \end{array}$$ (52)

After sorting out:


$$\begin{array}{cc} & \varepsilon_{c}\left(t_{i},t_{\text{s}},y_{skx}\right)-\frac{1}{2}\sigma_{c}\left(t_{i},y_{skx}\right)\left[J\left(t_{i},t_{i-1}\right)+J\left(t_{i},t_{i}\right)\right]+\sum_{ly=1}^{ky}\left\{\frac{1}{2}\mu \sigma_{c}\left(t_{i},y_{sky}\right)\left[J\left(t_{i},t_{i-1}\right)+J\left(t_{i},t_{i}\right)\right]\right\}\\ & =-\frac{1}{2}\sigma_{c}\left(t_{i-1},y_{skx}\right)\left[J\left(t_{i},t_{i-1}\right)+J\left(t_{i},t_{i}\right)\right]\\ & +\sum_{j=1}^{i-1}\left\{\frac{1}{2}\left[\sigma_{c}\left(t_{i},y_{skx}\right)-\sigma_{c}\left(t_{i-1},y_{skx}\right)\right]\left[J\left(t_{i},t_{j-1}\right)+J\left(t_{i},t_{j}\right)\right]\right\}\\ & -\sum_{j=1}^{i-1}\sum_{ly=1}^{ky}\left\{\frac{1}{2}\mu \left[\sigma_{c}\left(t_{i},y_{sly}\right)-\sigma_{c}\left(t_{i-1},y_{sly}\right)\right]\left[J\left(t_{i},t_{j-1}\right)+J\left(t_{i},t_{j}\right)\right]\right\}+\varepsilon_{\text{sh}}\left(t_{i},t_{\text{s}}\right)\\ & \varepsilon_{c}\left(t_{i},t_{\text{s}},y_{sky}\right)-\frac{1}{2}\sigma_{c}\left(t_{i},y_{sky}\right)\left[J\left(t_{i},t_{i-1}\right)+J\left(t_{i},t_{i}\right)\right]+\sum_{lx=1}^{kx}\left\{\frac{1}{2}\mu \sigma_{c}\left(t_{i},y_{skx}\right)\left[J\left(t_{i},t_{i-1}\right)+J\left(t_{i},t_{i}\right)\right]\right\}\\ & =-\frac{1}{2}\sigma_{c}\left(t_{i-1},y_{sky}\right)\left[J\left(t_{i},t_{i-1}\right)+J\left(t_{i},t_{i}\right)\right]\\ & +\sum_{j=1}^{i-1}\left\{\frac{1}{2}\left[\sigma_{c}\left(t_{i},y_{sky}\right)-\sigma_{c}\left(t_{i-1},y_{sky}\right)\right]\left[J\left(t_{i},t_{j-1}\right)+J\left(t_{i},t_{j}\right)\right]\right\}\\ & -\sum_{j=1}^{i-1}\sum_{lx=1}^{kx}\left\{\frac{1}{2}\mu \left[\sigma_{c}\left(t_{i},y_{slx}\right)-\sigma_{c}\left(t_{i-1},y_{slx}\right)\right]\left[J\left(t_{i},t_{j-1}\right)+J\left(t_{i},t_{j}\right)\right]\right\}+\varepsilon_{\text{sh}}\left(t_{i},t_{\text{s}}\right) \end{array}$$ (53)

Then,


$$\begin{array}{c} d_{x}=-\frac{1}{2}[J\left(t_{i},t_{i}\right)+J\left(t_{i},t_{i-1}\right)\{d_{y}\}=\frac{1}{2}\mu \left[J\left(t_{i},t_{i}\right)+J\left(t_{i},t_{i-1}\right)\right] \end{array}$$ (54)

$$\begin{array}{cc} & m_{lx}=-\frac{1}{2}\sigma_{c}\left(t_{n-1},y_{slx}\right)\left[J\left(t_{i},t_{i-1}\right)+J\left(t_{i},t_{i}\right)\right]\\ & +\sum_{j=1}^{i-1}\left\{\frac{1}{2}\left[\sigma_{c}\left(t_{j},y_{slx}\right)-\sigma_{c}\left(t_{j-1},y_{slx}\right)\right]\left[J\left(t_{i},t_{j-1}\right)+J\left(t_{i},t_{j}\right)\right]\right\}\\ & -\sum_{j=1}^{i-1}\sum_{ly=1}^{ky}\left\{\frac{1}{2}\mu \left[\sigma_{c}\left(t_{j},y_{sly}\right)-\sigma_{c}\left(t_{j-1},y_{sly}\right)\right]\left[J\left(t_{i},t_{j-1}\right)+J\left(t_{i},t_{j}\right)\right]\right\}+\varepsilon_{\text{sh}}\left(t_{i},t_{\text{s}}\right)\\ & m_{ly}=-\frac{1}{2}\sigma_{c}\left(t_{n-1},y_{sly}\right)\left[J\left(t_{i},t_{i-1}\right)+J\left(t_{i},t_{i}\right)\right]\\ & +\sum_{j=1}^{i-1}\left\{\frac{1}{2}\left[\sigma_{c}\left(t_{j},y_{sly}\right)-\sigma_{c}\left(t_{j-1},y_{sly}\right)\right]\left[J\left(t_{i},t_{j-1}\right)+J\left(t_{i},t_{j}\right)\right]\right\}\\ & -\sum_{j=1}^{i-1}\sum_{lx=1}^{kx}\left\{\frac{1}{2}\mu \left[\sigma_{c}\left(t_{j},y_{slx}\right)-\sigma_{c}\left(t_{j-1},y_{slx}\right)\right]\left[J\left(t_{i},t_{j-1}\right)+J\left(t_{i},t_{j}\right)\right]\right\}+\varepsilon_{\text{sh}}\left(t_{i},t_{\text{s}}\right)\\ & lx=1,2\cdots kx,ly=1,2\cdots ky \end{array}$$ (55)

### 4.3. Difference method for creep calculation

The difference of Eq (45) leads to:


$$\begin{array}{c} \varepsilon_{c}\left(t_{j},t_{s},y_{x}\right)=\varepsilon_{c}\left(t_{j-1},t_{s},y_{x}\right)\\ +\left\{\left[\sigma_{c}\left(t_{j},t_{s},y_{x}\right)-\mu \sigma_{c}\left(t_{j},t_{s},y_{y}\right)\right]-\left[\sigma_{c}\left(t_{j-1},t_{s},y_{x}\right)-\mu \sigma_{c}\left(t_{j-1},t_{s},y_{y}\right)\right]\right\}J\left(t_{i},t_{i}\right)\\ \varepsilon_{c}\left(t_{j},t_{s},y_{y}\right)=\varepsilon_{c}\left(t_{j-1},t_{s},y_{y}\right)\\ +\left\{\left[\sigma_{c}\left(t_{j},t_{s},y_{y}\right)-\mu \sigma_{c}\left(t_{j},t_{s},y_{x}\right)\right]-\left[\sigma_{c}\left(t_{j-1},t_{s},y_{y}\right)-\mu \sigma_{c}\left(t_{j-1},t_{s},y_{x}\right)\right]\right\}J\left(t_{i},t_{i}\right) \end{array}$$ (56)

After sorting out:


$$\begin{array}{c} \varepsilon_{c}\left(t_{i},t_{s},y_{x}\right)-J\left(t_{i},t_{i}\right)\sigma_{c}\left(t_{i},y_{x}\right)+\mu J\left(t_{i},t_{i}\right)\sigma_{c}\left(t_{i},y_{y}\right)\\ =\varepsilon_{c}\left(t_{i-1},t_{s},y_{x}\right)-J\left(t_{i},t_{i}\right)\sigma_{c}\left(t_{i-1},y_{x}\right)+\mu J\left(t_{i-1},t_{i}\right)\sigma_{c}\left(t_{i-1},y_{y}\right)+\varepsilon_{sh}\left(t_{i},t_{s}\right)\\ \varepsilon_{c}\left(t_{i},t_{s},y_{y}\right)-J\left(t_{i},t_{i}\right)\sigma_{c}\left(t_{i},y_{y}\right)+\mu J\left(t_{i},t_{i}\right)\sigma_{c}\left(t_{i},y_{x}\right)\\ =\varepsilon_{c}\left(t_{i-1},t_{s},y_{y}\right)-J\left(t_{i},t_{i}\right)\sigma_{c}\left(t_{i-1},y_{y}\right)+\mu J\left(t_{i-1},t_{i}\right)\sigma_{c}\left(t_{i-1},y_{x}\right)+\varepsilon_{sh}\left(t_{i},t_{s}\right) \end{array}$$ (57)

Then,


$$\begin{array}{c} d_{x}=-J\left(t_{i},t_{i}\right)\\ d_{y}=\mu J\left(t_{i},t_{i}\right) \end{array}$$ (58)

$$\begin{array}{c} m_{lx}=\varepsilon_{c}\left(t_{i-1},t_{s},y_{slx}\right)-J\left(t_{i},t_{i}\right)\sigma_{c}\left(t_{i-1},y_{slx}\right)+\sum_{ly=1}^{ky}\mu J\left(t_{i},t_{i}\right)\sigma_{c}\left(t_{i-1},y_{sly}\right)+\varepsilon_{sh}\left(t_{i},t_{s}\right)\\ m_{ly}=\varepsilon_{c}\left(t_{i-1},t_{s},y_{sly}\right)-J\left(t_{i},t_{i}\right)\sigma_{c}\left(t_{i-1},y_{sly}\right)+\sum_{lx=1}^{kx}\mu J\left(t_{i},t_{i}\right)\sigma_{c}\left(t_{i-1},y_{slx}\right)+\varepsilon_{sh}\left(t_{i},t_{s}\right)\\ lx=1,2\cdots kx,ly=1,2\cdots ky \end{array}$$ (59)

At this time, trapezoidal method and difference method can still be solved by Eq (35).

### 4.4. Example analysis

**Fig 9:**
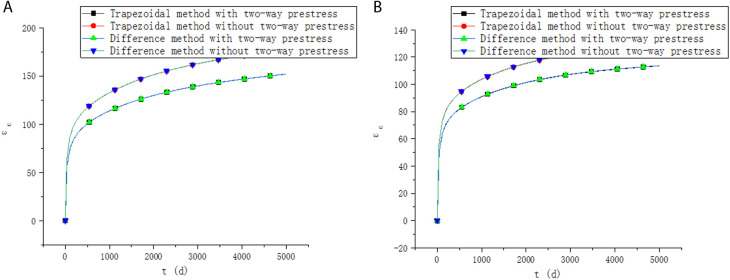
Strain of upper edge. (a) X direction. (b) Y direction.

**Fig 10:**
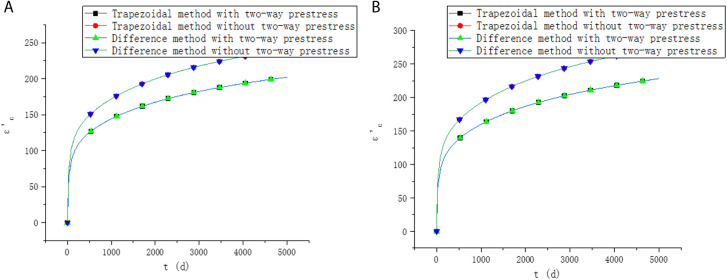
Strain of lower edge. (a) X direction. (b) Y direction.

**Fig 11:**
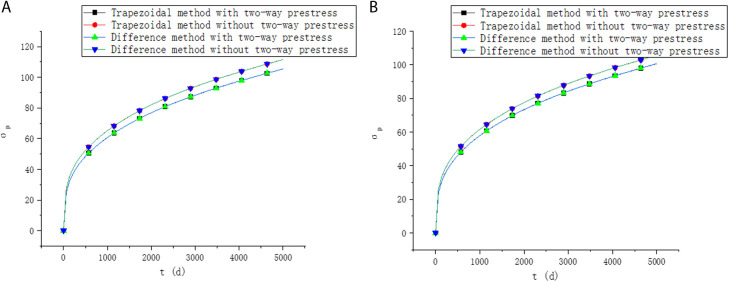
Prestress loss. (a) X direction. (b) Y direction.

In order to further explain the influence of two-way prestressed reinforcement on the long-term performance, a two-way prestressed concrete with 1000mm×1000mm×1000mm size is assumed in this paper. The concrete strength grade is C50. Only prestressed reinforcement is provided in the x and y directions, the reinforcement ratio is $\rho_{x}=1\;\%,\rho_{y}=1.2\;\%$, the ultimate strength is $f_{x}=930MPa,f_{y}=1860MPa$, and the initial tensile stress is $\sigma_{x}=0.65f_{x},\sigma_{y}=0.70f_{y}$. The distance from the gravity center of the prestressed reinforcement to the upper edge of the section is $y_{x}=600mm,y_{y}=700mm$. The relative humidity is 80%. Poisson’s ratio is 0.2. The long-term performance with and without two-way prestress is shown in Figs 9–11.

It can be seen from Figs 9–11 that the calculation results of the two methods in this paper are similar. When considering the influence of two-way prestress, the strain and prestress loss are lower than those without considering the influence of two-way prestress. The creep development of two-way prestressed members is slower than that of one-way prestressed members. This may primarily be attributed to the following factors. First, by arranging prestressing tendons in two orthogonal directions, two-way prestressing enhances the structural restraint, limiting the free deformation of concrete. Especially in the early stage of creep, the internal stress distribution becomes more uniform, deformation is more restricted, and the progression of creep slows down. Second, the stress field in two-way prestressed structures is more balanced, reducing local stress concentrations, which helps suppress the development of microcracks and delays the onset of creep. In addition, the lateral confinement provided by transverse prestressing effectively slows down the creep process in concrete, functioning similarly to stirrups by mitigating transverse cracking and strain propagation that would otherwise occur in uniaxial prestressed systems. Under the influence of two-way prestressing, deformations in different directions mutually constrain each other, weakening the coupling effect and reducing the mutual influence between stress transfer and deformation. As a result, two-way prestressed structures can effectively slow down the creep rate and enhance the long-term stability of the structure.

## 5. Conclusion

In this paper, based on the principle of concrete creep superposition, the trapezoidal method and the difference method are developed to refine the long-term performance analysis of prestressed concrete. The main conclusions are as follows:

(1) The proposed trapezoidal and difference methods enable accurate calculation of long-term deformation in one-way prestressed concrete members, as validated against existing refinement methods and experimental results.
(2) Compared with conventional creep refinement approaches, the methods presented in this study significantly reduce computational workload, enhance efficiency, and provide a reliable theoretical foundation for creep analysis in large-scale structures.
(3) The creep calculation method is further extended to two-way prestressed concrete, addressing limitations in current codes and research. The results indicate that the development of creep in two-way prestressed members is slower than that in one-way members.

Future work will focus on integrating the proposed methods into numerical analysis tools for large-scale structures, and validating them with additional long-term experimental data, especially for two-way prestressed concrete. Further research will also consider environmental influences such as temperature and humidity, aiming to develop a more accurate and practical creep prediction framework for engineering applications.

## Supporting information

S1 File Data. (ZIP)
